# Eco-friendly remediation of tetracycline antibiotic from polluted water using waste-derived surface re-engineered silica sand

**DOI:** 10.1038/s41598-023-37503-4

**Published:** 2023-08-12

**Authors:** Osamah Al-Hashimi, Khalid Hashim, Edward Loffill, Ismini Nakouti, Ayad A. H. Faisal, Tina Marolt Čebašek

**Affiliations:** 1 Babylon Water Directorate, Babylon, 51001 Iraq; 2 Faculty of Engineering and Technology, School of Civil Engineering and Built Environment, Liverpool John Moores University, Liverpool, L3 3AF UK; 3 Built Environment and Sustainable Technology Research Institute, Liverpool John Moores University, Liverpool, L3 3AF UK; 4 Department of Environmental Engineering, College of Engineering, University of Baghdad, Baghdad, Iraq

**Keywords:** Environmental sciences, Chemistry

## Abstract

A new green reactive adsorbent (calcium ferric oxide silica sand (CFO-SS)) made from wastepaper sludge ash and ferric ions was synthesised and shown to remove tetracycline antibiotics (TC) from contaminated water effectively. The synthesised sand was dried at 95 °C, and a series of batch and fixed bed experiments were performed to determine the optimum operating conditions. Results showed that the adsorption capacity of the CFO-SS increases with the concentration gradient between the solid and liquid phases. 0.3 g of the new adsorbent was proven sufficient to remove more than 90% of the TC at a pollutant dose of 50 mg/L in 50 mL of simulated groundwater with an agitation speed of 200 rpm for 3 h. The adsorption isotherm followed the Langmuir isotherm model, with a loading capacity of 21.96 mg/g at pH 7, while the Pseudo second-order model best described the absorption kinetics. The adsorption mechanisms proposed included electrostatic interaction, intraparticle diffusion, hydrogen bonding, and cation-π interactions. Characterisation investigations revealed that the newly precipitated oxides on silica sand play an essential role in TC adsorption support. In fixed-bed experiments, it was discovered that reducing the flow rate and inflow concentration of TC and increasing the sorbent mass significantly extended the lifetime of the produced sorbent in the packed column. The measured breakthrough curves were best fit with the Adams-Bohart and the Clark models, as they provided the highest square root number (R^2^) values. Finally, considering the efficacy of CFO-SS in TC adsorption performance, it can be noted that the novel synthesised reactive material is an efficient and environmentally friendly material for TC removal, and it presents a potential solution to resolving the challenge of TC-rich groundwater.

## Introduction

According to the United Nations (UN) World Water Development Report of 2018, an estimated six billion people all over the world will endure acute water resource depletion by 2050. The primary causes of this water tension encompass population and economic growth, climate change and global warming, and exaggerated use of water supplies in the industrial and agricultural sectors^1^. Groundwater is considered a crucial resource for freshwater, and sustainable management of these hidden resources is considered very important for future generations, as well as for socioeconomic and environmental impacts^2^. Thousands of tons of human-made chemicals are used daily by societies, all of which have the potential to reach water resources through direct discharge from wastewater treatment plants, landfills, and land use by human and animal waste on farms^3–5^. The presence of pharmaceutical pollutants in the surface and groundwater resources has been documented^6–8^. According to the findings of^9^; TC is one of the most used pharmaceutical antibiotics; it is usually used in human and veterinary medicine. Moreover, TCs are added to animal nutrition in several countries, notably the United States, at subtherapeutic concentrations to serve as growth accelerators^10^. Researchers documented that TC has been present in groundwater, surface water, and even in drinking. The presence of TC in water resources may increase the dangers posed by microbiological populations developing drug resistance and producing even more harmful degradation by-products. Therefore, removing TC is indeed essential^11,12^. Groundwater is usually treated using a broad range of techniques. The pump and treat technique is a popular method where polluted groundwater is piped to treatment plants and treated using a variety of techniques, including electro-deposition, chemical precipitation, and adsorption. After treatment, water is repumped to the subsurface or discharged to the nearest sewer system. However, this method has many limitations, including the high cost of pumping and well drilling, as well as the low permeability of some subsurface sections, which limits the effectiveness of the pump and treat method. Finally, this method may cause changes in the hydraulic gradient of the groundwater in the treatment area^13–16^. Other methods are used to treat the groundwater, such as in-situ flushing and in-situ air sparging. The most promising, efficient, and cost-effective method is the permeable reactive barriers (PRBs)^17–19^. In the permeable reactive barrier, a continuous wall or curtain of replaceable, semi-replaceable or permanent reactive materials is placed in a barrier transversely against the direction of the contamination plume; this wall can also be placed in a funnel and gate configuration which ensures that all the groundwater will pass through the (gate) which consist of the reactive media of the (PRBs) and that will achieve better remediation to the groundwater. Selecting the suitable configuration of the PRB depends on the site characteristics and the efficiency of the reactive media^20,21^.

Zero-valent iron (ZVI) and nanoscale ZVI are examples of many materials that are employed as reactive media in PRBs^22–24^; in addition, zeolite^25^, granular activated carbon (GAC)^26,27^, ion exchangers^28^ are also regular reactive materials. Despite the use of those reactive materials, investigators are currently looking for cost-effective, environmentally friendly reactive media that use by-product materials in their composition^13^. In this paper, a novel calcium ferric oxide silica sand (CFO-SS) has been synthesised through an optimisation process. As a concept of environmental sustainability, the wastepaper sludge ash (WPSA) has been used as a rich source for calcium ions to react with the ferric ion for the creation of a nanolayer of calcium ferric oxides; therefore, the primary argument for carrying out this study is to assess how effectively this sorbent can adsorb TC, which is prevalent in groundwater.

Sand that has been coated with calcium ferric oxides is composed of calcium and ferric ions; this sand can be regarded as a particularly reactive component. The sand here is created using the co-precipitation method. Accordingly, this paper aims to: (I) synthesise/optimise a novel sorbent from a by-product waste. (II) Use the synthesised sorbent for the adsorption of TC from water through a series of batch tests and one-dimensional fixed bed column tests. (III) Identify the predominant mechanism by characterisation analysis.

## Materials and methods

**Figure 1:**
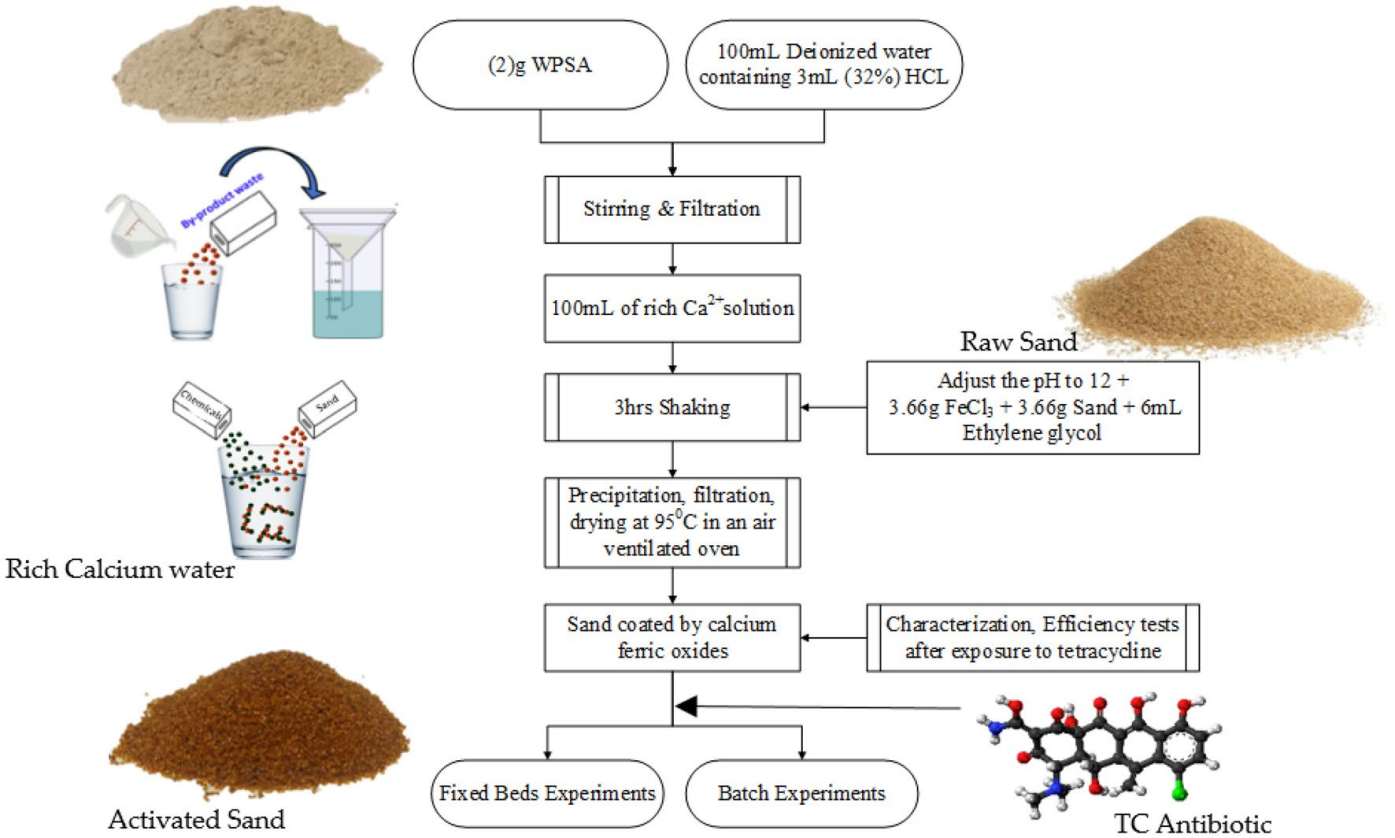
Block diagram for the processes of sand coated by calcium ferric oxides (CFO-SS).

Wastepaper sludge ash (WPSA) has been supplied by SAICA PAPER UK Ltd, the physical properties of the used WPSA are 13.34 µm mean diameter, pH 12.31, SG 2.5, and bulk density 561 kg/m^3^. Industrial sand, supplied by Liverpool John Moores University (LJMU), with a mean diameter of 1013 µm, SG of 2.685, a porosity of 0.37 and hydraulic conductivity of 4.719 × 10^–1^ cm/s. TC powder has been supplied from Merck, UK, along with ethylene glycol (purity ≥ 99%), FeCl_3_ (purity: 97%), HCL (32%) and NaOH. The following steps have been followed to synthesise the sand coated by calcium ferric oxides, which are illustrated in Fig. 1.

Two grams of WPSA were added to 100 mL of deionised containing (v/v) 1.5 mL of 32% HCl. After 3 h of vigorous stirring at 200 rpm at room temperature, the solution was filtered through Whatman filter papers grade 1.0 to separate the solids and collect calcium (Ca^2+^) ions dissolved in the clear filtrate solution. The pH value of the solution of (Ca^2+^) was adjusted to 12. Then, a measured amount (3.66 g) of FeCl_3_ (To achieve a molar ratio of Ca^2+^/FeCl_3_ of 1:0.75) was added to the solution. 3.66 g of quartz sand, which achieve a dosage of 1 Sand: 1 FeCl_3_, added to the mixture. A suitable volume (6 mL) of ethylene glycol was decanted in a glass flask and shaken at 200 rpm for 3 h to form a coating layer of calcium ferric oxides on the surface of the sand. The mixture was then filtered using Whatman filter paper grade (1) to separate the sand from the solution. The resulting sand was then dried in an air-ventilated oven at 95 °C for 12 h. Sand is then kept in a suitable glassed bottle for further use.

### Adsorption experiments and batch tests

The reactivity of the calcium ferric oxides coated silica sand (CFO-SS) has been investigated through a series of batch experiments; the optimum operation environment which assures the best removal efficiency has been examined, and the procedure for batch experiments was the same as^29–31^.

A benchtop Visible Spectrophotometer (UV-Spectrophotometer) type HACH DR3900 has been utilized to measure the concentration of TC. Furthermore, the wavelength for TC has been calculated by measuring the peak absorbance for three random concentrations of TC. Additionally, a calibration curve for TC measurements has been established using a range of TC concentrations (10–200 mg/L). In the batch experiments, sets of 250 mL glass flasks were employed, with each flask containing 50 mL of deionized water (17.5 MΩ). To ensure proper contact between the reactive material (CFO-SS) and the pollutant (TC), a specific mass of 0.1 g of CFO-SS was added to each flask, which was then shaken using a benchtop shaking incubator type Labnet 222-DS. After a shaking period of 3 h, the clear solution of the treated water was obtained by filtering out the suspended reactive media particles using Whatman filter paper grade (5). The batch studies aimed to investigate the optimum operating environment for achieving the best adsorption. These studies examined the effect of different initial concentrations of TC (10–200 mg/L) over time (10–240 s), as well as the impact of pH (2–12) on the initial dose of CFO-SS (0.05–0.5 g) and the influence of agitation speed (0–250 rpm). Results were subsequently verified through Fourier transform infrared analysis (FT-IR), SEM, and XRD techniques, which were employed to analyze the raw silica sand, CFO-SS, and the synthesized materials after their interaction with TC.

### Fixed bed column experiments

**Figure 2:**
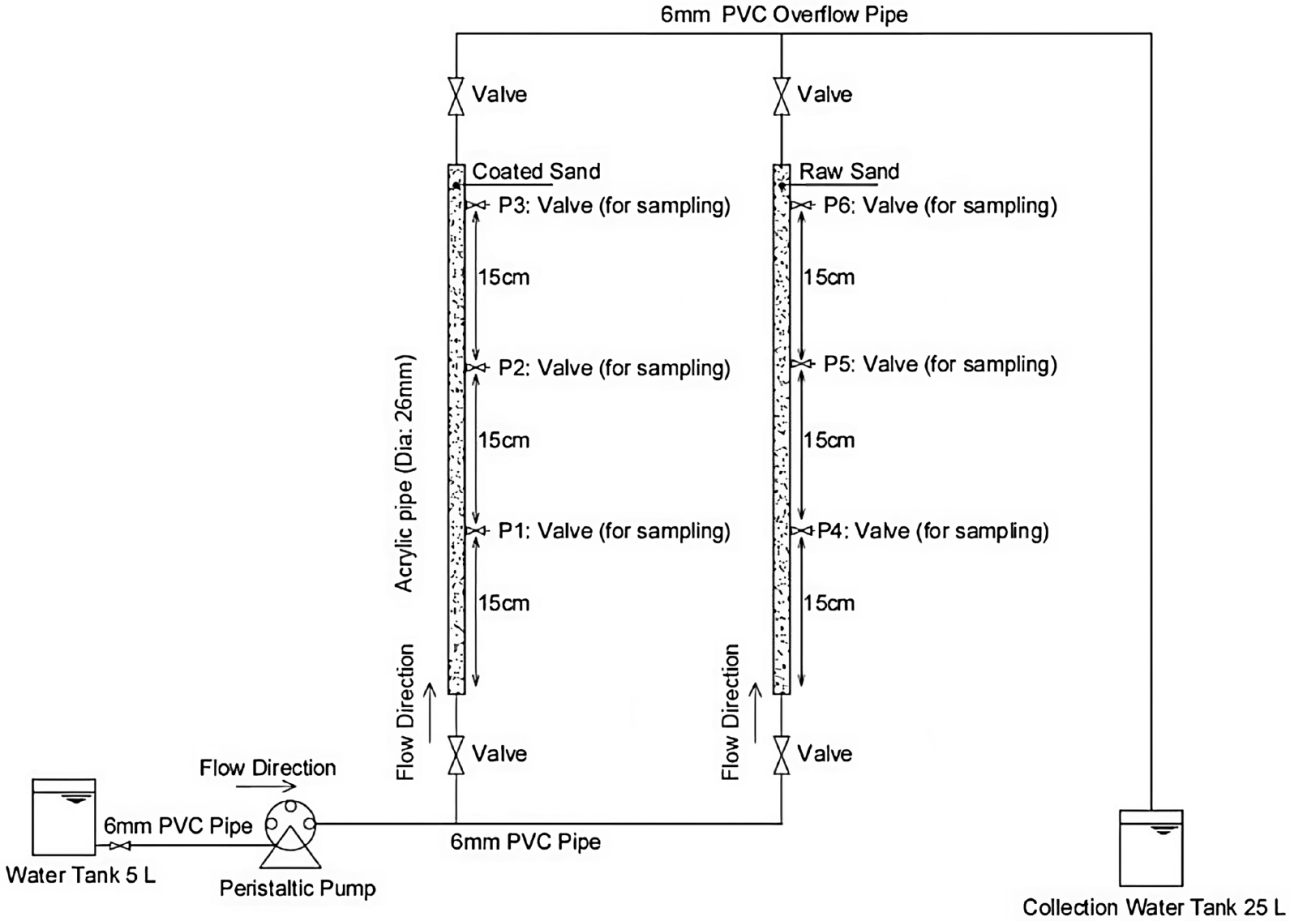
One-dimensional underground lab scale simulator model.

Fixed bed column tests were carried out to imitate the spread of contaminants in a one-dimensional flow, which closely resembles the actual scenario in operating a permeable reactive barrier, where the contaminated water flows upward. The WATSON MARLOW, peristaltic pump model 520S, was utilised to circulate the contaminated water from a tank, with flow rates of (1.58, 4.75, and 9.5 mL/min), as shown in Fig. 2. These are the lowest flow rates that could be achieved by the pump, ensuring the movement of groundwater in a creeping/laminar flow with Reynold's number of less than 10. The effectiveness of the reactive media was evaluated by monitoring the normalised contaminant concentration in the effluent throughout the operation until the reactive media was exhausted. Three key factors that affect the adsorption capabilities, mass transfer zone, and bed utilisation value in a fixed bed column are the influent flow rate, influent concentration, and bed height. These elements play a crucial role in the overall performance of the permeable reactive barrier system^32^.

### Adsorption isotherms

The interaction between a substance and a solid phase in an aqueous environment or porous medium at a constant temperature and pH is shown by the adsorption isotherm curve. When the concentration of the adsorbate in the solution equals the concentration at the interface, after a sufficient length of time, the equilibrium between the adsorbate and adsorbent is achieved. Over time, several models for the adsorption isotherm have been developed using diverse theoretical and empirical methods. These isotherms include:

#### Freundlich isotherm

An empirical relation established by Freundlich in 1909 describes the maximum amount of gas that can be absorbed per unit mass of a solid under pressure. The Freundlich adsorption isotherm is a graph matching the solute quantity at the solid surface with that in the surrounding aquatic environment. It's characterised by Eq. (1), which expresses the adsorption as a function of adsorbate concentration as follows:

1 $${q}_{e}={K}_{f}{C}_{e}^{1/n}$$

where (${K}_{f}$) and ($n$) are Freundlich for intensity and capacity.

The Freundlich isotherm indicates that sorbet impurities are strictly proportional to their concentration in low amounts. Therefore, they drop as pollutants accumulate on the surface of the reactive media; this scenario occurs when Freundlich isotherm is prevalent.

#### Langmuir model

According to Langmuir, each adsorptive molecule should be adsorbed to a distinct binding domain on the adsorbent. Adsorption continues until all these sites are occupied, resulting in further saturation. Each active binding spot on the adsorbent can only accept one adsorbate molecule at a time. The equation for the Langmuir isotherm model is as shown in Eq. (2)

2 $${q}_{e}=\frac{{q}_{m}b{C}_{e}}{1+{C}_{e}}$$

In this equation, the maximum adsorption capacity is ($${q}_{m})$$, while the adhesion of the contaminant to the reactive component is given by ($$b$$).

### Breakthrough curves and pollutants transport equation

A partial differential equation that governs space and time is used in the theory of contaminants transport in porous media. This theory takes into consideration four distinct processes. The first one is advection, which describes a situation in which a substance flows in the same direction as convection while also following the direction of water flow. The dispersion process, which is induced by the heterogeneity of contaminants in the environment, is the second process; in other words, some pollutants move through the environment at a faster rate than others. The kinetic equation describes the chemical reaction that directs the flow of pollutants in groundwater clearly and accurately. Finally, contaminants adsorbed to the soil may spend time in either the solid phase or the mobile water phase. Adsorption is the process by which contaminants are cleared from the environment. The advection–dispersion equation, which is written as follows in Eq. (3), describes these components best.

3 $$\frac{\partial m}{\partial t}=\frac{\partial (nC)}{\partial t}=-(\frac{\partial F}{\partial x}+\frac{\partial F}{\partial y}+\frac{\partial F}{\partial z})\mp r$$

In the above equation, the term $(r)$ refers to the change in mass per unit volume (m) of the pollutants due to the reactions inside the aquifer.

In Eq. (4); The advection–dispersion is expressed for a one-dimensional flow assuming a constant dispersion coefficient and constant porosity in space and time (which would be equal to one in a saturated medium):

4 $${R\frac{\partial C}{\partial t}=D}_{x}\frac{{\partial }^{2}C}{\partial {x}^{2}}-{V}_{x}\frac{\partial C}{\partial x}$$

where ($R$) is the retardation factor, which indicates the effect of contaminants being retarded down while they are transported downwards. The "breakthrough curve" refers to the relationship between contaminant concentration and time. This "breakthrough curve" is an essential tool for designing and maximising the sorption in a field-scale PRB by relating the data that was obtained from laboratory fixed bed columns to the breakthrough curves for the field scale. The breakthrough curve will have the shape of an S when contaminants are introduced continuously and constantly; the point on this curve that is preferable, known as the breakthrough point, will have an outlet concentration of contaminants that is equivalent to the desired concentration in the environment. The following is a brief description of a variety of empirical and theoretical models that can be used to anticipate breakthrough curves:

#### Bohart–Adams model

The primary objective of column experiments is to figure out the relationship between concentration and time and to get the breakthrough curve, as well as the maximum amount of adsorbent needed. Results will be used to plan an adsorption column at full size. One of the models used for this purpose is the Bohart–Adams model, which is based on the theory of the rate of surface reaction^33^. The model is based on the following suggestions^34^:

- The concentration at relatively low levels could be described using this model;
- When $t\to \infty ;{q}_{0}\to {N}_{0}$ with saturation concentration;
- The rate of adsorption is limited by the ambient mass transfer;

The formula for the Bohart–Adams model is illustrated in Eq. (5) below:

5 $$\frac{C}{{C}_{0}}=\frac{1}{1+\mathrm{exp}(K{N}_{0}\frac{Z}{U}-K{C}_{0}t)}$$

where ${C}_{0}$ and $C$ are the initial and instantaneous pollutant concentrations in mg/litre of solution, respectively. The rate of change in mass per unit of time is represented by the constant kinetic $K$ (L/g/min). For congestion, ${N}_{0}$ is the concentration in millimoles per litre. Column bed depth is defined by $Z$ (cm). Time ($t$) is expressed in minutes, whereas flow rate ($U$) is expressed in centimetres per second.

#### Belter–Cussler–Hu model

It is a new semi-empirical fixed bed model, and it is shown in Eq. (6) below:

6 $$\frac{C}{{C}_{0}}=1+\mathrm{erf}\left[\frac{\left(t-{t}_{0}\right)\mathrm{exp}\left(-\mathcal{o}\left(\frac{t}{{t}_{0}}\right)\right)}{\sqrt{2}\mathcal{o}{t}_{0}}\right]$$

where (erf) is the error function, ($t$) is the column retention time, (${t}_{0}$) is the time when the concentration of the effluent is half the concentration of the feed water, and ($\mathcal{o}$) is the standard deviation, which is a measure of the gradient of the breakthrough curve in its straight.

#### Yoon–Nelson model

In this model, each adsorbate's decreasing possibility is proportionate to its breakthrough retention on the adsorbent. This approach is demonstrated by the following Eq. (7):

7 $$\mathit{ln}\frac{C}{{C}_{F}-C}={K}_{YN}t-{t}_{1/2}{k}_{YN}$$

where ${K}_{YN}$ represents the Yoon–Nelson rate constant.

#### Clark model

The breakthrough curves of Clark were based on the mass transfer concept and the Freundlich isotherm. As Eq. (8); Clark developed the following breakthrough curves:

8 $${\left(\frac{C}{{C}_{0}}\right)}^{n-1}=\frac{1}{1+A.{e}^{-rt}}$$

where $n$ is for the exponent of the Freundlich isotherm, $A$ refers to the parameters of the kinetic equation, and $r$ refers to the rate constant.

## Results and discussion

### Batch experiments/operational conditions

The proper operational conditions have been studied by the batch experiments; these conditions are the initial pH, sorbent dose, the effect of contact time, agitation speed and the initial concentration of the TC (C_0_). In all experiments, 250 mL flasks were used, and 50 mL of contaminated water with varying TC concentrations (10, 50, 100, 150, and 200 mg/L) were tested; 0.1 g of the synthesised (CFO-SS) was applied with an agitation speed of 200 rpm. After the 3 h, the water was filtered using grade (5) filter paper to separate any impurities. Ultraviolet–visible spectrophotometer (UV) type (HACH DR3900) has been used to detect the final concentration of TC (C_e_). The amount of TC adsorbed by the (CFO-SS) reactive media has been calculated using Eq. (9), while the removal efficiency has been calculated using Eq. (10)^35,36^:

9 $${q}_{e}=({C}_{0}-{C}_{e})\frac{V}{m}$$

10 $$R({\%})=\frac{({C}_{0}-{C}_{e})}{{C}_{0}} \times 100$$

Where's, $${(q}_{e}$$) is the amount of adsorbate loaded on the adsorbent (mg/g), ($${C}_{0}, {C}_{e}$$) are the initial and final concentration of the adsorbate (mg/L), (V) is the volume of aqueous solution (L), and (m) is the mass of adsorbate (g). the (%) removal efficiency of the contaminant is ($$R)$$.

The time required to achieve the equilibrium state for (10, 50, 100, 150 and 200 mg/L) concentrations has been investigated. All experiments have been performed in pH (7), (CFO-SS) dose of 0.1 g for each 50 mL of contaminated water with TC, agitation speed (200 rpm). Results showed that the adsorption rate was fast in the first 60 min and then slowed down due to the reduction in the available sites on the adsorbate and the occupation of most of the sites by TC molecules. Results showed that 180 min were sufficient to reach the maximum TC loading on the (CFO-SS) particles. While increasing the concentration of TC in solution, the removal efficiency was reduced from 90% when the concentration of TC was 10 mg/L to 21% when the concentration was 200 mg/L, which relates to the fact that most of the available sites on the (CFO-SS) are being occupied and there are no available sites for more TC molecules. Another important factor in the batch experiments has been investigated, the pH value of the aqueous solution, a range of aqueous pH (2–12) have been investigated, TC initial concentration (C_0_) was 50 mg/L, and (CFO-SS) dose was 0.1 g, at 200 rpm agitation speed. Results showed that increasing the pH value of the aqueous solution enhanced the removal efficiency; the maximum removal was at pH (10), and after that, the removal efficiency reached an equilibrium state. To justify this behaviour. A Zeta Potential test has been performed using the solid addition method (ΔpH) by a (0.15) g of (CFO-SS), 0.01 M NaCl, agitation speed at 200 rpm for 24 h. Results revealed that the charge of the (CFO-SS) is negative below 5 and positive above it. TC is a broad-spectrum antibiotic which consists of 59.45% of (C), 5.44% (H), 6.30% (N) and 28.80% of (O), with amphoteric, phenolic, and alcoholic properties^37^. Owing to the presence of a dimethylammonium group, a phenolic diketone moiety, and a tricarbonyl system, TC exists mostly as cationic (${\mathrm{TCH}}_{3}^{+}$) at pH less than 3.3, zwitterionic (${\mathrm{TCH}}_{2}^{\pm }$)) between pH 3.3–7.7, and anionic (${\mathrm{TCH}}^{-}$) and (${\mathrm{TCH}}_{2}^{-}$) at a pH greater than 7.7^38,39^. The higher electrostatic attraction between TC and the (CFO-SS) is due to the high intensity of the (+) charge on the adsorbate, which will attract with the (-) charge of the (${\mathrm{TCH}}_{2}^{-}$). The effect of sorbent dose and the optimum dose that remove most of the pollutant in a 50 mL of polluted water having 50 mg/L TC, pH 7, agitation speed of 200 rpm for 180 min. The sorbent dose has increased from 0.05 to 0.5 mg. Results showed that a dose of 0.3 g (CFO-SS) can remove 91.8% of the TC, while the removal efficiency was 53.4% with a dose of 0.05 g. After 0.3 g, an equilibrium has been reached because most of the TC molecules have been adsorbed, and an equilibrium is achieved between the sorbent and the aqueous environment. The speed of agitation is an important factor^40^ that affects the adsorption of TC on the engineered (CFO-SS), at pH (7), 0.3 g of sorbent for 50 mL containing 50 mg/L TC, the agitation speed has been altered from (0) to (250) rpm, results reviled that the optimum agitation occurs at (200)rpm. This is due to the high agitation that caused disruption to the pollutant; it seems that the agitation at 200 rpm provided the proper contact time between the pollutant and the adsorbate, accordingly. The removal efficiency at 200 rpm was 91.8%, while it was 81.1% and 90.7% when the agitation was 150 rpm and 250 rpm, respectively. The batch experiments have been extensively detailed in the author's previous article^41^

### Sorption isotherm

**Figure 3:**
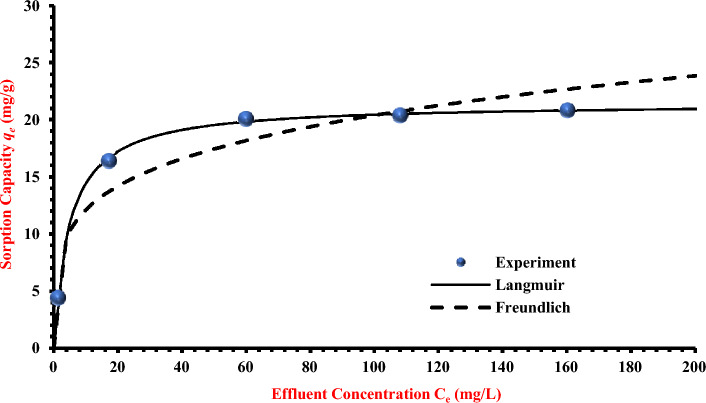
The rate constant of isotherm models with statistical measurements of TC sorption onto the artificial sand covered with calcium ferric oxides.

The adsorption isotherm is used to represent the distribution of contaminants on the solid phase at equilibrium and to compute the maximal adsorption capacity and the adsorbent's affinity. These isotherms are necessary relationships to characterise the interaction in the packed bed between the contaminants and the coated sand. The acquired sorption results of TC adsorption by the synthesised coated sand are fitted using nonlinear versions of Freundlich and Langmuir models by employing the "solver" option nonlinear regression in Microsoft Excel Software 365. Table 1; shows the model constants, the sum of squared errors (SSE), and R^2^. Results suggest that the Langmuir model can better represent the obtained measurements than the Freundlich interpretation. According to the values of R^2^ and SSE (0.995 and 0.202), respectively), the Langmuir model appears to be more suited for measurement formulation than the Freundlich model. The matching between the measurements and the Langmuir model, on the other hand, is described in Fig. 3; the highest capacity and affinity constant values for the interaction of TC with present sorbent were 21.450 mg/g and 0.204 L/mg, respectively, which is very close to the maximum experiments (*q*_e_) (21.96 mg/g).

**Table 1:** Isotherms models for the adsorption of TC.

Model	Parameter	Value
Freundlich	*K*_*f*_ (mg/g) (L/mg)^1/n^	7.222
	*N*	4.437
	*R*^*2*^	0.880
	*SSE*	24.299
Langmuir	*q*_*m*_ (mg/g)	21.450
	*b *(L/mg)	0.204
	*R*^*2*^	0.995
	*SSE*	0.202

### Characterisation of sand coated with calcium ferric oxides

X-ray fluorescence analysis and XRF analysis have been performed to investigate the chemical composition of the used WPSA; results showed that the predominant oxide forming the WPSA is the CaO which represents about 34% of the total composition, as shown in Table 2; below.

**Table 2:** XRF analysis for WPSA.

Chemical consentient	Calcium oxide (lime)	Chlorine	Sulfur Trioxide	Silicon dioxide (silica)	Aluminium trioxide	Sodium oxide	Phosphorus pentoxide	Titanium dioxide
Empirical Formula	CaO	Cl	SO_3_	SiO_2_	Al_2_O_3_	Na_2_O	P_2_O_5_	TiO_2_
Chemical Composition (w/w%)	34.004	8.775	3.144	3.111	3.071	2.872	1.572	0.804

**Figure 4:**
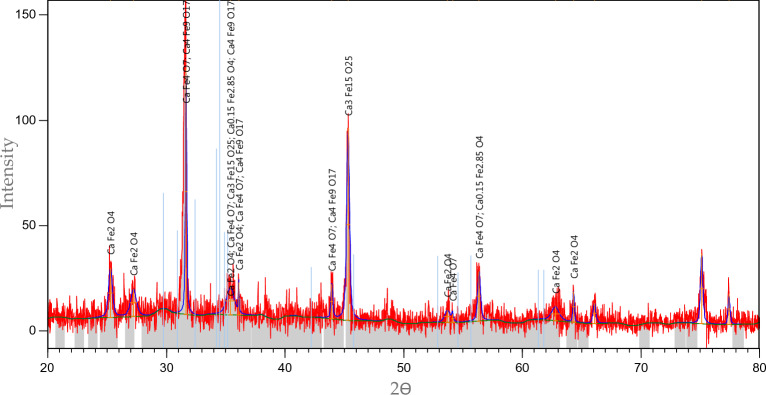
The XRD analysis for the coated sand.

The XRD spectral test for the (CFO-SS) has been performed, as illustrated in Fig. 4. Results have been analysed using the PANalytical/X'Pert HighScore Plus software for XRD powder diffraction measurements; it is revealed that calcium ferric oxides have appeared on the sand after the modification process. Measurements have been compared with the (Joint Committee on Powder Diffraction Standards (JCPDSs)) and found that the silica oxide is the major ingredient responsible for the presence of peaks. The X'Pert HighScore Plus software revealed the synthesis of (CaFe_4_O_7,_ Ca_3_Fe_15_O_25,_ Ca_0.15_Fe_2.85_O_4,_ CaFe_2_O_4_ and Ca_4_Fe_9_O_17_). These compounds are responsible for the adsorption of TC as it turned the raw sand into a reactive media have the ability to catch the pollutants in an aqueous environment due to the intensification of Fe ion^42^ and Ca ion^43^.

**Figure 5:**
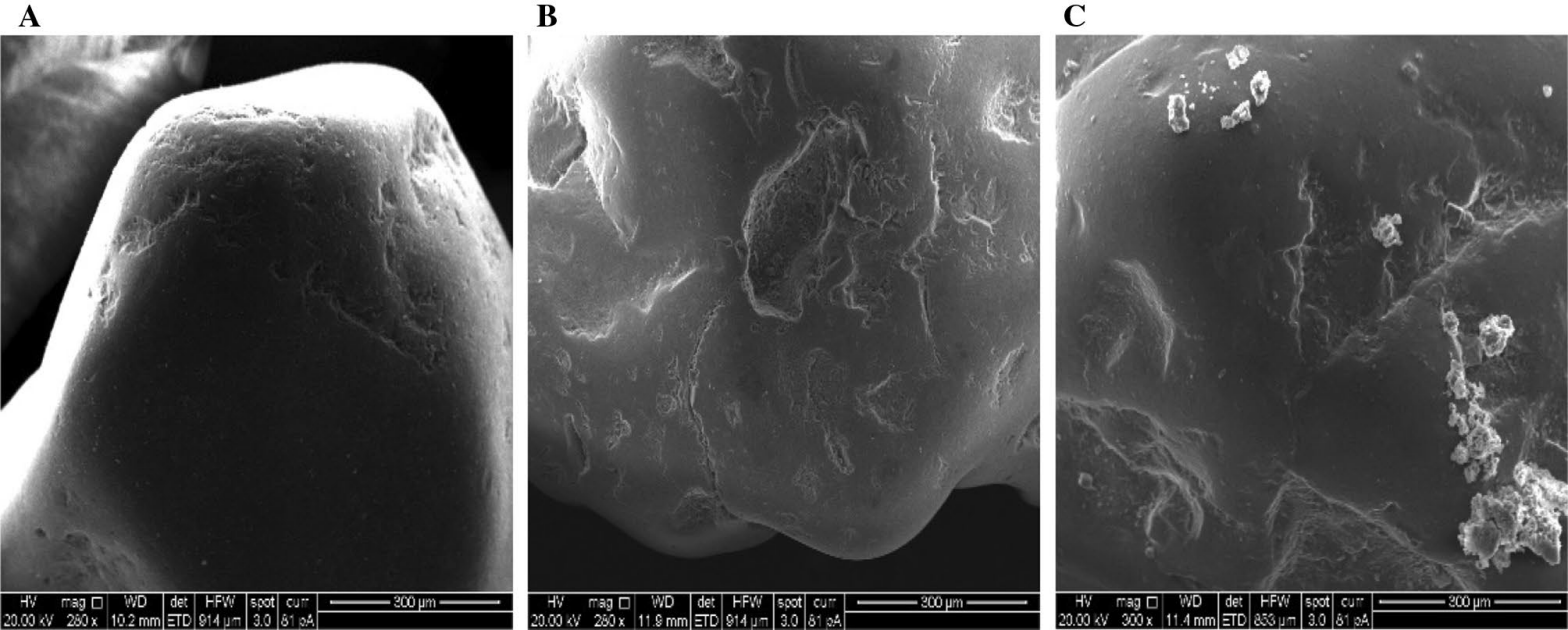
SEM (scanning electron microscopic) pictures of (**A**) raw sand, (**B**) CFO-SS before interaction with TC, and (**C**) CFO-SS after interaction with TC.

The morphology of raw, synthesised sand before and after its combination with TC has been investigated using FEI Inspect-S (SEM) variable vacuum (0.1–30 kV range); results in Fig. 5 revealed a smooth surface of the sand, the coated sand increased surface roughness due to the immobilising of calcium ferric oxides on the sand surface, this led to the attraction of TC to the coated sand.

**Figure 6:**
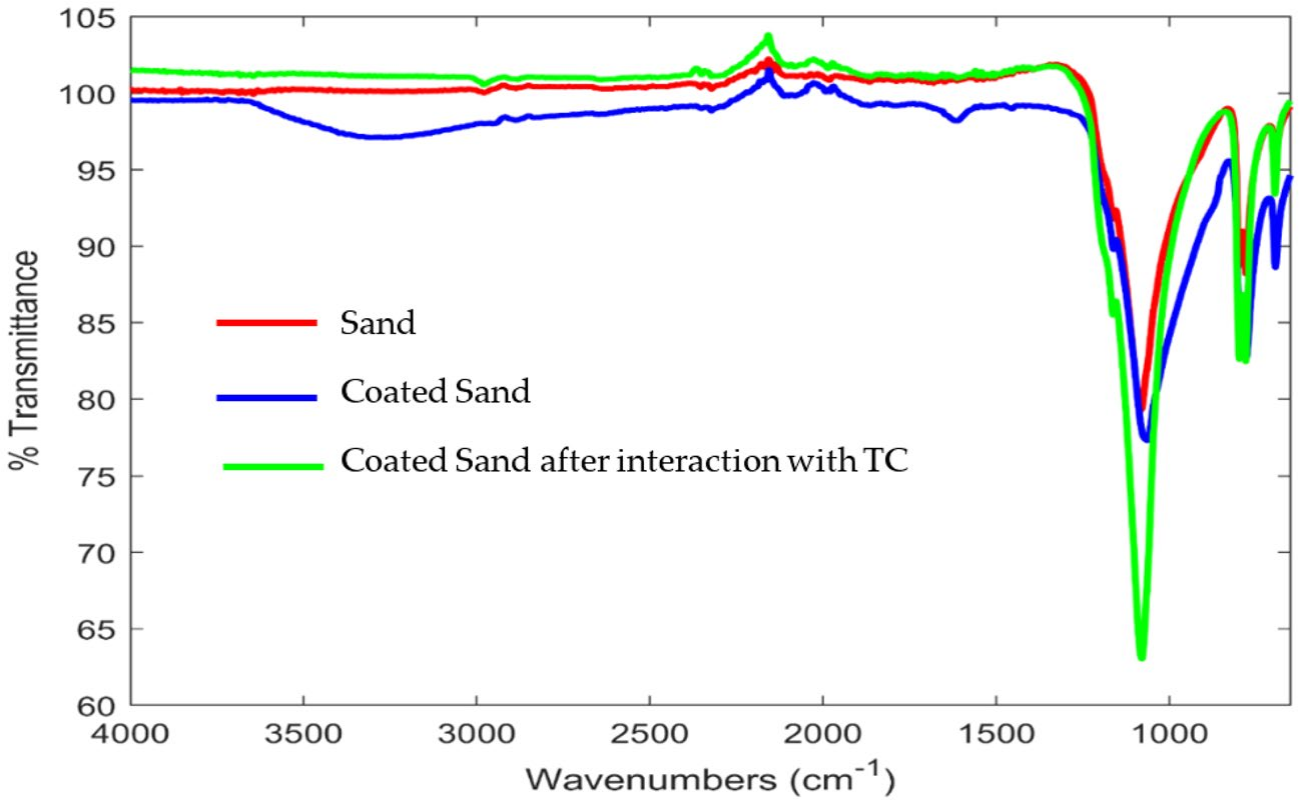
FT-IR characterisation of sand and composite sorbent before and after exposure to TC.

Infrared spectroscopy investigates the interaction of infrared radiation with sand, coated sand, and coated sand after TC adsorption. Results showed that the IR adsorption for these research samples follows the typical IR adsorption as shown in Fig. 6; in other words, the presence of Si–O vibration bending occurring at 777 cm^−1^, 779 cm^−1^ and 693 cm^−1^ reveals the presence of quartz sand^19,29,44^. The stretching vibrations of (OH) groups can cause high-intensity absorption bonds^29^. The peak of sand coated with calcium ferric oxides at 3375 cm^−1^ is due to the stretching mode of the OH group, stretching vibration of a hydrogen bond or formation of interlayer water molecules. The peaks at 1162 cm^−1^ are attributed to the C–O tensile vibrations and C–O rings resulting from the deformation of C–C–H and C–O–H^45^. Asymmetric and symmetric stretching vibrations of aliphatic C–H were observed at 2957 cm^−1^^45^. The Si–O–Si bond is seen on distinct 1086, 797, 695, 1069, 799, 1082, and 797 absorption bands. Silanol groups (Si–OH) which appear at wavenumber 846 cm^−1^, could be activated on the surface of the grains of the matrix^46^, which could cause the attachment of TC chains to the surface of grains of sand with the formation of hydrogen bonds of the type Si–O–H⋅⋅⋅O–H. Furthermore, the presence of a (C–O–Si) bond at a wavenumber of 1164 cm^−1^ can result from binding interactions between the coating matrix and the matrix (silane Si–OH groups). Based on the FTIR analysis, it is indicated that presence of a wide spectrum of functional groups. This analysis concluded that all media producing different band spectra would give different adsorption intensities due to different surface functional groups.

### Influence of fixed bed operation conditions

The purpose of the column tests was to simulate the transport of contaminants in a one-dimensional flow, which closely resembles the actual functioning of a permeable reactive barrier. The contaminated water was pumped using a WATSON MARLOW peristaltic pump model 520S with flow rates of 1.58 mL/min, 4.75 mL/min, and 9.5 mL/min. These flow rates were the minimum that could be achieved by the pump while maintaining a creeping/laminar flow with Reynold's number of less than 10. The performance of the reactive media was evaluated by monitoring the effluent's normalised contaminant concentration over time until the reactive media was depleted. The results of these tests provide valuable insight into the efficacy of permeable reactive barriers in the treatment of contaminated water.

#### Influence of influent flowrate

**Figure 7:**
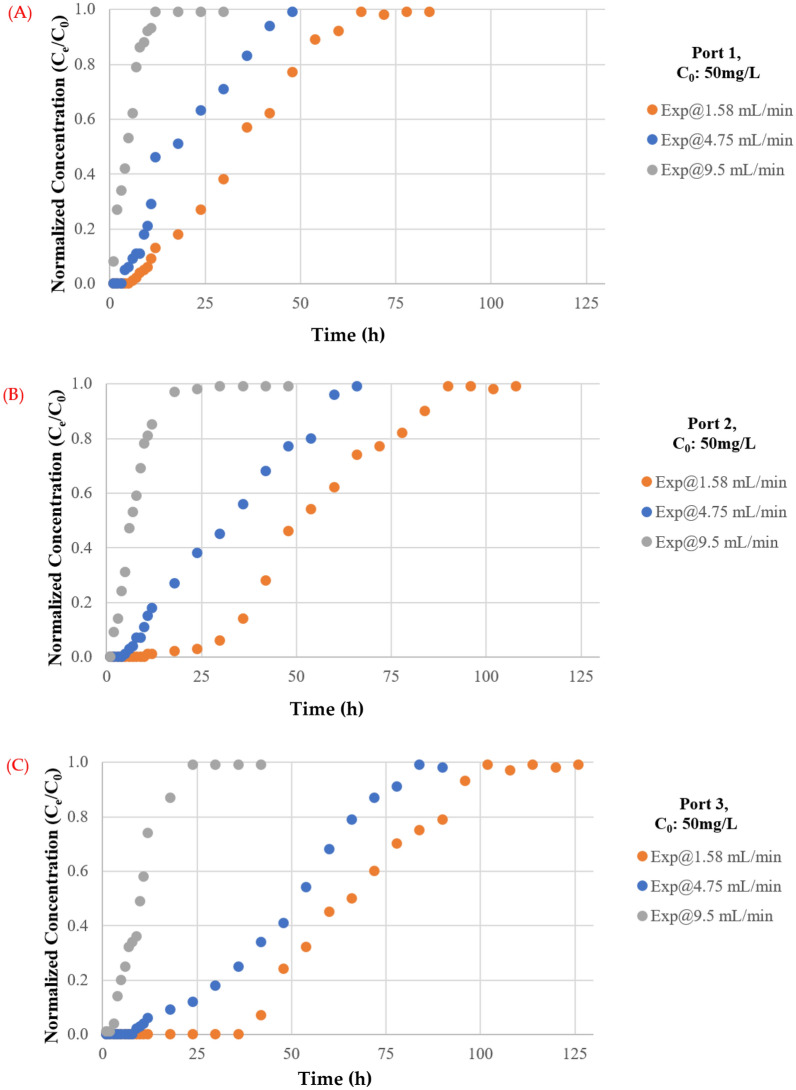
Measurements of normalized concentration (C_e_/C_0_) with time at same initial concentration of TC and different discharges for (**A**) Port 1, (**B**) Port 2 and (**C**) Port 3.

The effect of flowrate on the mass of TC maintained in the barrier has been investigated using flowrates of 1.57, 4.75, and 9.5 mL/min. The plotting of breakthrough curves based on measurements of normalised concentrations with varying flow rates at ports 1, 2, and 3 are shown in Fig. 7. It is obvious that a high discharge can shorten the time required for the breakthrough and boost the steepness of the curve because contaminated water leaves before reaching equilibrium, as predicted. This phenomenon is caused by insufficient antibiotic residence time in the column at a high flowrate^47^, restricting antibiotic molecule diffusion into the pores of the synthesized coated sand. Furthermore, the adsorbed TC on the adsorbent may be desorbed to the aquatic environment as an effect of the high flowrates which will reduce the removal efficiency^48^. However, the higher flowrate had a shorter mass transfer zone. The lowest flowrate had the highest adsorption of total antibiotic. As water velocity increased, adhesion between solute and sorbent may decrease, resulting in a noticeable decrease in sorption effectiveness. In addition, desorption of certain molecules of a sorbet contaminant is possible, particularly for loose and reversible bonds with the sorbent. As a result, the concentration of TC in the effluent might rapidly increase, resulting in an early breakthrough time. The low flowrate aided pollutant adsorption in the fixed bed, which is consistent with prior research such as^49–52^.

#### Influence of pollutant concentration

**Figure 8:**
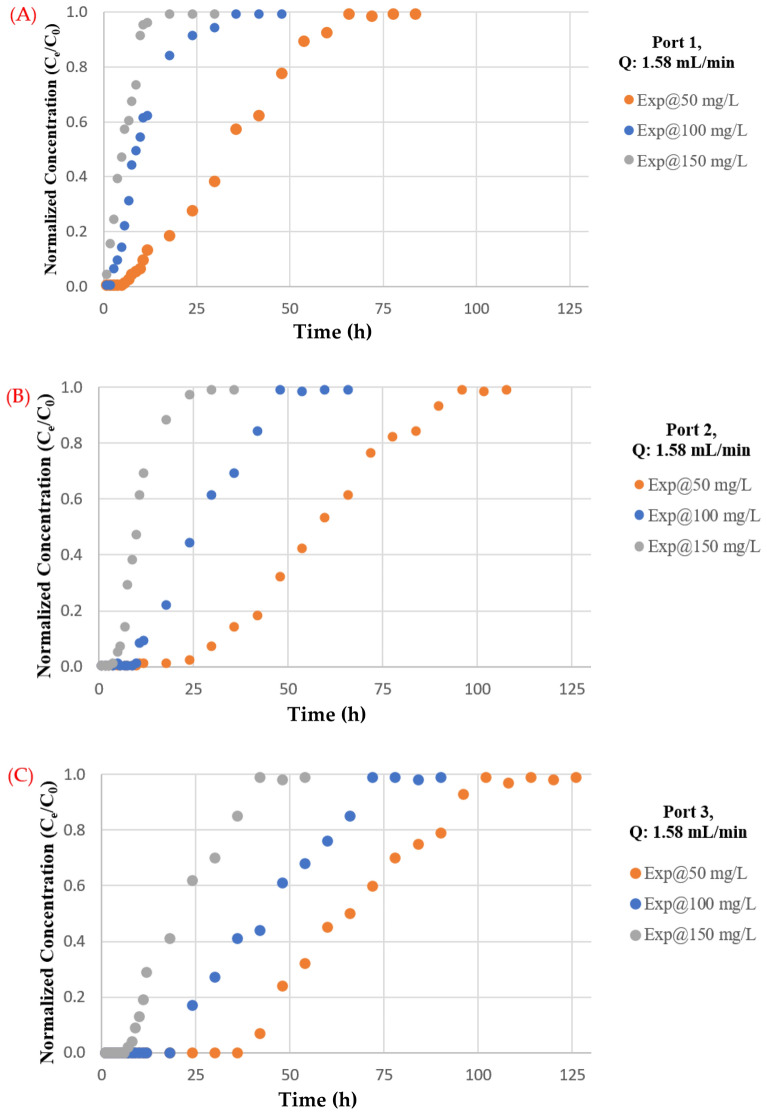
Measurements of normalized concentration (Ce/C0) with time at same discharge and different initial TC concentrations for (**A**) Port 1, (**B**) Port 2 and (**C**) Port 3.

The effect of feed antibiotic initial concentration depicted in Fig. 8. As the influent antibiotic concentration increased from 50 to 150 mg/L in port (3), the exhaust time for TC decreased from (102) to (36) h. A greater driving force for antibiotic mass transfer resulted from a higher influent concentration^31,53^. On the contrary, when the concentration of the influent antibiotic decreased, the breakthrough curves rise later, indicating a wider transfer zone and slow intraparticle diffusion process^54^. It should be noted that the adsorption capability increases with the concentration of influent antibiotics. This is because a larger concentration gradient resulted in faster mass transfer due to an increased surface diffusion coefficient. Moreover, when the concentration of influent antibiotics increases, so does the overall amount of antibiotics adsorbed. At flow rates of 1.58, 4.75, and 9.5 mL/min for ports 1, 2, and 3, the effect of influent concentration on the front of the breakthrough curves is examined. Due to a slower adsorption, the curve is less noticeable at lower entry concentrations; nevertheless, as the concentration rises, the steepness of the curve increases, and the bed acquires saturated more quickly. Additionally, a notable fall in the magnitude of the mass transfer coefficient might occur together with a decrease in the concentration gradient. The transmission of the contaminant front will be delayed as a result for this reduction, and the "exhaustion time" will shorten as the inflow concentration rises, resulting in a reduction in the amounts of contaminant that are adsorbed inside the bed. The results from the influent concentration effect have been confirmed by comparing it with the results from literature such as^55,56^.

#### Influence of the bed depth

**Figure 9:**
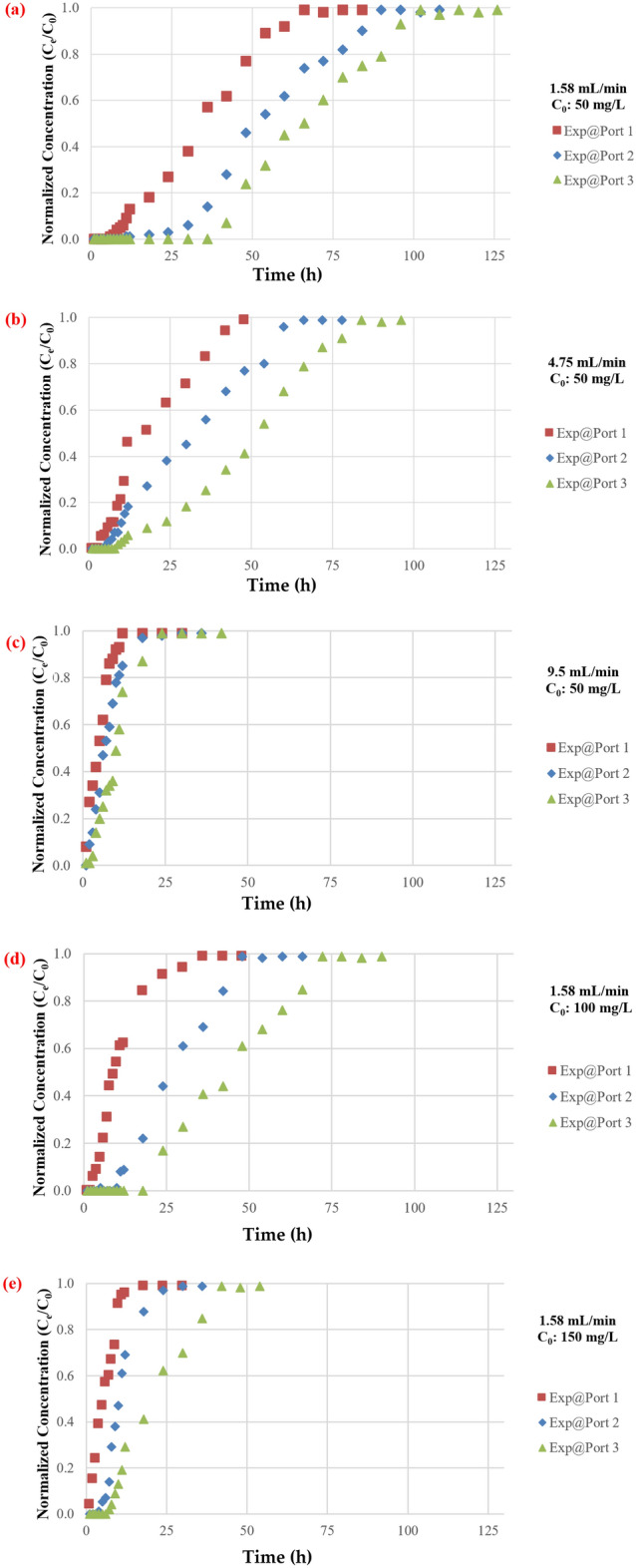
Measurements of normalized concentration (C_e_/C_0_) time at same discharge and initial TC concentrations for (**a**) Q: 1.58 mL/min & C_0_: 50 mg/L, (**b**) Q: 4.75/min & C_0_: 50 mg/L; (**c**) Q: 9.5 mL/min & C_0_: 50 mg/L; (**d**) Q: 1.58 mL/min & C_0_: 100 mg/L; (**e**) Q: 1.58 mL/min & C_0_: 150 mg/L.

Increasing the bed depth from 15 to 30, and 45 cm resulted in a substantial decrease in TC concentration in the barrier for all flowrates and baseline concentration values used in this research as shown in Fig. 9. This might be due to the contaminated fluid remaining in the bed for an extended length of time, which will increase the absorption process due to the increase of the available surface area of the adsorbent and increase in the contact time^57^. However, barrier's functionality diminished with time due to the saturation of the bed with sorbet pollutant, this finding have been confirmed by^58,59^.

#### Behaviour similarity with conventical fixed bed column models

Four traditional adsorption models were used for simulating adsorbate transport in a fixed bed column and employed to fit the adsorption experiments data as a comparison with an expected model; these models are Bohart–Adams Model, Yan Model, Belter–Cussler–Hu Model and Clark Model.

**Figure 10:**
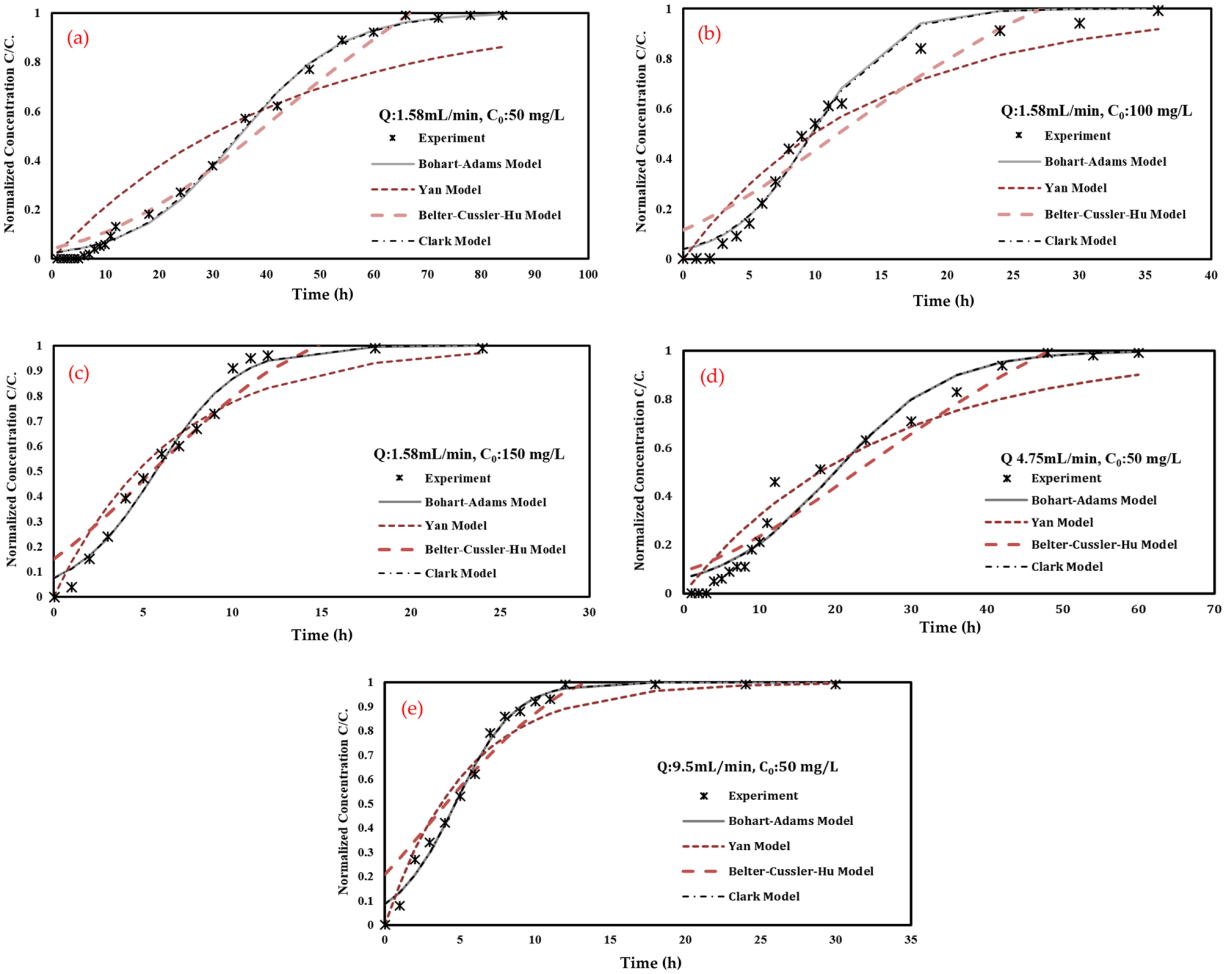
Breakthrough curves for sorption of TC onto CFO-SS at port 1 at different TC concentrations and discharges (**a**) Q: 1.58 mL/min; C_0:_ 50 mg/L, (**b**) Q: 1.58 mL/min; C_0:_ 100 mg/L, (**c**) Q: 1.58 mL/min; C_0:_ 150 mg/L, (**d**) Q: 4.75 mL/min; C_0:_ 50 mg/L, (**e**) Q: 9.5 mL/min; C_0:_ 50 mg/L.

As shown in Table 3 and Fig. 10, in all ports, experimental data seems to best fit with the Adams–Bohart model and Clark model as it gave the largest square root number (R^2^) (for port 1: R^2^ were 0.9953, 0.9652, 0.9755 and 0.9945 for Adams–Bohart, Yan, Betler-Cussler-Hu and Clark models, respectively). This assumes that the equilibrium is not instantaneous and the adsorption rate is proportional to the concentration of adsorbate.

**Table 3:** Values of quantitative evaluation metrics for fixed bed models at various column outlets.

Model name	Parameter	C_0_ = 50 mg/L	C_0_ = 100 mg/L	C_0_ = 150 mg/L	Q = 1.58 mL/min	Q = 4.75 mL/min	Q = 9.5 mL/min
Port 1 fixed bed experiments							
Bohart–Adams model	Kc_0_	0.10	0.33	0.44	0.10	0.14	0.51
	kN.Z/u	3.64	3.25	2.51	3.64	2.71	2.37
	SSE	0.02	0.05	0.04	0.02	0.10	0.02
	R^2^	0.9953	0.9824	0.9808	0.9953	0.9670	0.9887
Yan model	R^2^	0.9652	0.9673	0.9648	0.9652	0.9808	0.9722
Belter–Cussler–Hu model	R^2^	0.9755	0.8933	0.9100	0.9755	0.9385	0.9008
Clark model	R^2^	0.9945	0.9818	0.9808	0.9945	0.9666	0.9241
Port 2 fixed bed experiments							
Bohart–Adams model	Kc_0_	0.08	0.15	0.51	0.09	0.09	0.42
	kN.Z/u	4.92	4.19	5.16	4.66	3.07	2.90
	SSE	0.01	0.03	0.02	0.03	0.05	0.02
	R^2^	0.9979	0.9919	0.9943	0.9945	0.9867	0.9928
Yan model	R^2^	0.8926	0.9346	0.9183	0.9096	0.9790	0.9684
Belter–Cussler–Hu model	R^2^	0.9813	0.9658	0.8889	0.9714	0.9750	0.8684
Clark model	R^2^	0.9970	0.9890	0.9940	0.9937	0.9865	0.9923
Port 3 fixed bed experiments							
Bohart–Adams model	Kc_0_	0.08	0.09	0.56	0.08	0.08	0.33
	kN.Z/u	5.48	4.08	3.55	5.48	4.26	3.31
	SSE	0.04	6.01	0.06	0.04	0.01	0.03
	R^2^	0.9929	0.9912	0.9817	0.9929	0.9970	0.9883
Yan model	R^2^	0.8780	0.9497	0.9590	0.8780	0.9142	0.9619
Belter–Cussler–Hu model	R^2^	0.9734	0.9682	0.9417	0.9734	0.9912	0.8576
Clark model	R^2^	0.9919	0.9900	0.9599	0.9919	0.9960	0.9882

These models are widely used to describe the breakthrough curve. Adams–Bohart's model well described the transport of TC in the column. It is also noticeable that the values of (kC_0_) increase with increasing the TC influent concentration and discharge rate and, in most cases, decrease with the increase of bed depth. The results also indicated that the adsorption of TC, in the column, decreases with the increase of the influent concentration of TC, the flow rate and the decrease of the bed.

### Potential mechanism of adsorption

**Figure 11:**
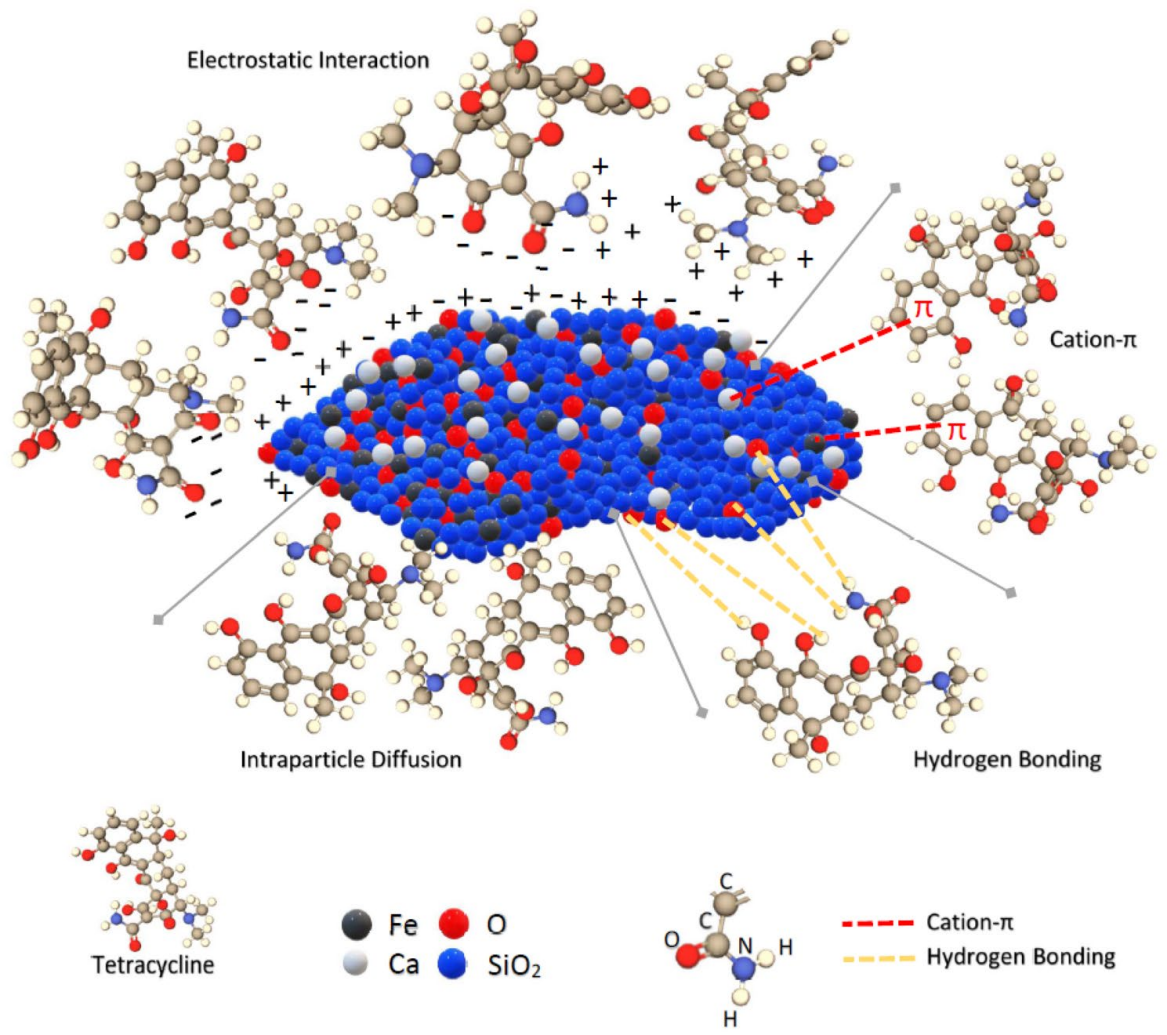
The potential mechanism for the adsorption of TC by CFO-SS.

The adsorption of TC on the synthesised (CFO-SS) layer is influenced by four interactions; these are the electrostatic interaction, cation-π interaction, H-bonding and intraparticle diffusion. The characterisation tests performed on the raw sand, CFO-SS, before and after the adsorption gives an obvious clue to the adsorption mechanism; in the beginning, the XRD test for the raw sand and for the coated sand proves the synthesise of calcium ferric oxides on the sand surface, the enrichment of sand surface by compounds rich with calcium and ferric ions in addition to the oxygen are responsible for the catchment of TC from the aqueous environment. TC has (3) pKa values at (3.3, 7.7 and 9.7), which means that TC can chemically interact at different pH values by donating its protons. The Zeta potential test proves that the surface charge of the (CFO-SS) is negative under (5) and positive over (5). This will enable the CFO-SS to interact with (H +) and (O−) ions on the TC, which leads to an electrostatic attraction between the TC and the (CFO-SS) surface. The SEM proved the change in surface morphology of the coated sand due to the formation of calcium ferric oxides and the drying of the engineered sand, which led to an increase the surface roughness. This led the molecules of TC to interlock within the coated sad grooves by the intraparticle diffusion; the intraparticle diffusion kinetics proves this phenomenon. This research examines the difference in the FTIR spectra of CFO-SS before and after the adsorption of TC. The obtained spectra, including the FTIR spectrum of TC, are presented in Fig. 11. However, the iron and calcium elements in the Fe-OH and Ca-OH likely provided an uninhabited orbital for lone pair of electrons and π-bonding electrons in TC functional groups (hydroxyl, carbonyl, and amino groups) and benzene ring forming TC-Fe, TC-Ca complexes to strengthen the cation-π interaction. This explanation completely agrees with the fact that the iron and calcium elements demonstrated that the antibiotic-metal complex could facilitate the elimination of antibiotics^60,61^.

## Conclusions

The composite sorbent (CFO-SS) was shown to be a good, efficient material for groundwater loaded by TC since it could be contaminated by TC antibiotics with a maximum adsorption capacity of 21.96 mg/g. Modified, engineered sand could be used as a reactive media as it has excellent hydraulic conductivity if used in a permeable reactive barrier. In this research, the sorption isotherm experiments were performed at pH 7, sorbent mass = 0.1 g/50 mL, shaking speed = 200 rpm, room temperature, duration = 3 h for C_o_ = 10–200 mg/L. The Langmuir model accurately fitted with the adsorption measurements. According to the characterisation investigations, surface complexation, electrostatic, cation-π, and h-bonding interaction are responsible for the TC removal. Continuous (fixed bed experiments) suggests that the breakthrough curves had a classical S-shape, with early and late breakthrough, reflecting nonideal transport. For TC adsorption, raising the bed height increase the exhaustion time of the reactive media, resulting in a larger mass transfer zone. TC concentration dropped as bed height increased. The overall adsorbent effective surface area increased with the increment of bed height. For future studies, it is recommended to investigate the ability of new reactive materials that are made in total from by-product waste. This will achieve the concept of treating the waste by waste and will serve the environment through the reuse of waste instead of its disposal.

## Data Availability

The datasets generated and/or analysed during the current study are not publicly available due it is part of a PhD project in which the authors have to adhere to the ethical guidelines and regulations set forth by Liverpool John Moores University. However, the dataset will be available from the corresponding author upon reasonable request.
